# Casual effect of ulcerative colitis on chronic heart failure: results from a bidirectional Mendelian randomization study

**DOI:** 10.1186/s12876-025-03671-y

**Published:** 2025-02-20

**Authors:** Yuzhou Chu, Jianhua Li, Li Gong, Sheng Shao, Hao Chen, Pengfei He, Juntao Yan

**Affiliations:** 1 Department of Tuina, Yueyang Integrated Traditional Chinese and Western Medicine Hospital, Shanghai University of Traditional Chinese Medicine, Shanghai, 200437 P. R. China; 2 Department of Cardiovascular, Yueyang Integrated Traditional Chinese and Western Medicine Hospital, Shanghai University of Traditional Chinese Medicine, Shanghai, 200437 P. R. China

**Keywords:** Ulcerative colitis, Mendelian randomization, Heart failure

## Abstract

This study aimed to detect the causal effect of ulcerative colitis (UC) on heart failure. A bidirectional two-sample Mendelian randomization (MR) analysis was performed. The causal impact of UC on heart failure was determined via MR by performing a genome-wide association study in which 4 UCs descending from European ancestors were set as individual exposures. The inverse-variance weighted (IVW) method was used as the main method, and 4 other methods were set as assistant parameters. Susbequently, the MR results were combined with meta-analysis results. The MR Egger method was employed to investigate pleiotropy. The leave-one-out method was utilized for sensitivity analysis. Furthermore, a reverse-directional study was conducted. There was evidence of the causal effect of UC on heart failure in MR estimates using 4 UC datasets. The IVW method revealed that the odds ratio (OR) = 1.03, 95% confidence interval (CI) = 1.01–1.06, *P* = 0.0441 when the first UC dataset was used; OR = 1.03, 95% CI = 1.01–1.05, *P* = 0.0445 when the second UC dataset was used; OR = 2046, 95% CI = 1.37-3.05E + 06, *P* = 0.0409 when the third UC dataset was used; and OR = 8.12E + 04, 95% CI = 29.09-2.27E + 08, *P* = 0.0052 when the fourth UC dataset was used. A meta-analysis of 4 MR studies revealed that UC had a statistically significant causal effect on heart failure (OR = 1.03, 95% CI = 1.01–1.05; *P* = 0.0074). Reverse MR analysis revealed that heart failure did not have a causal effect on UC. There was no pleiotropy. This MR study demonstrated that UC had a causal effect on heart failure and that there was no reverse causal effect.

**Supplementary Information:**

The online version contains supplementary material available at 10.1186/s12876-025-03671-y.

## Introduction

Ulcerative colitis (UC) is a chronic relapsing and remitting mucosal inflammatory bowel disease (IBD) of the colon. Crohn’s disease and UC are two types of inflammatory bowel disease. UC commonly starts in the rectum and extends through the entire colon. The global incidence rate of UC is increasing. For example, the incidence of UC in the United Kingdom is 12.6/100,000 person-years [1]. Furthermore, UC can cause a considerable economic burden, including costs of 8.1 to 14.9 billion dollars in the United States [2]. A genome-wide association study (GWAS) revealed that many genes may increase the occurrence of UC. Mendelian randomization (MR) is an increasingly popular method for inferring causality in epidemiological research via genome-wide association studies (GWASs) [3]. Compared with other observational designs, MR analysis is characterized by its ability to aid in clinical trial design [4]. In recent years, many MR studies have demonstrated that UC has causal relationships with many other diseases and symptoms, such as psoriasis and psoriatic arthritis [5], rheumatoid arthritis [6], depression [7], Graves’ disease [8] and nonalcoholic fatty liver disease [9].

Heart failure is a complex myocardial dysfunction syndrome with typical insufficient echocardiographic parameters of the left ventricular ejection fraction [10]. It is rare in young individuals, and its morbidity increases with age. Heart failure is common among elderly individuals. There are many risk factors for heart failure [11] – most notably, myocardial injury caused by congenital heart disease, ardiomyopathies, myocarditis, or cardiotoxicity caused by the abuse of antineoplastic drugs. Many antineoplastic drugs cause cardiac injury and therefore have adverse cardiovascular effects, resulting in acute or delayed onset of heart failure [12]. In addition to myocardial injury factors, other diseases or factors can increase the occurrence and development of heart failure. There are even sex differences in the incidence of heart failure [13]. Despite the large number of risk factors and molecular targets that have been identified to date, there are a lack of satisfactory prevention and treatment strategies for heart failure. Therefore, it is crucial to identify additional new risk factors and mechanisms. Inflammation plays important roles in the progression of cardiovascular disease. Accumulating evidence suggests that there is a significantly increased risk of heart failure in patients with inflammatory bowel disease [14, 15]. However, the biological evidence supporting the genetic connection between these two diseases is limited from the perspective of MR analysis. In this study, MR was employed to investigate the causal effect UC on heart failure.

## Materials and methods

### Study type and design

**Fig. 1:**
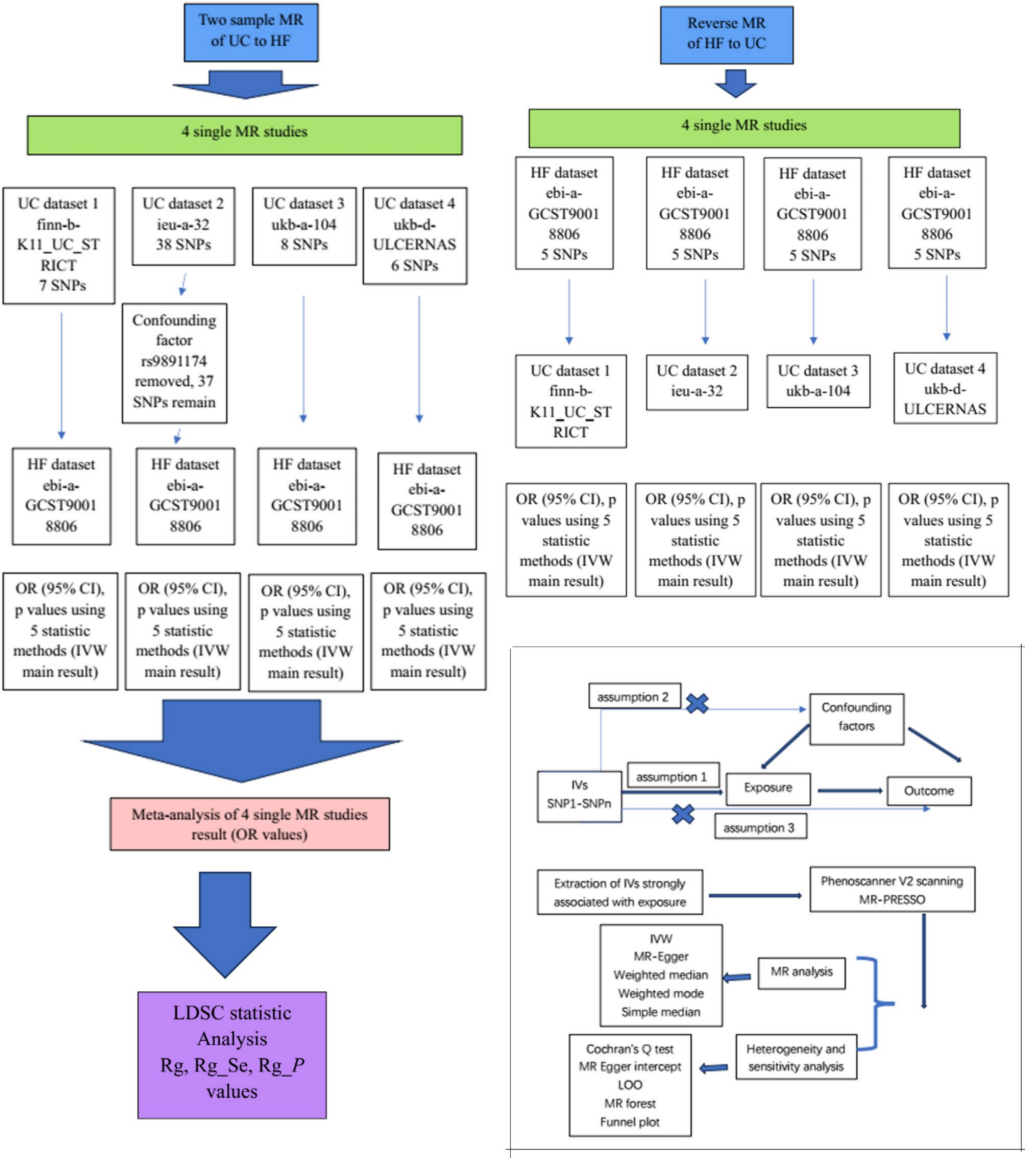
The flow chart of this comprehensive MR study (HF: heart failure, UC: ulcerative colitis)

This was a comprehensive two-sample MR analysis. MR analysis relies on the following key assumptions [16]: genetic variation is strongly linked to exposure, genetic variants should not be considered confounders, and genetic variants should be related to outcomes only via exposure. The data used herein were previously published data from studies that obtained ethics approval. Therefore, ethics approval was not required for the current study. This study was conducted in accordance with the STROBE-MR guidelines [17]. The flowchart of this study is shown in Fig. 1.

### Data

The genetic associations included in this MR analysis were obtained from GWASs [18]. The permissions were obtained to access the GWAS database(s). The dataset selection criteria were as follows: (1) the dataset included a disease-affected group and a control group. (2) to avoid duplication of the patient group as much as possible, the UC patient dataset was selected from those published within the past ten years without duplication in their respective years and from different database sources; and (3) more than 10,000,000 SNPs. Four UC datasets of self-reported or meet strict definition were included as the exposure datasets, whereas 1 chronic heart failure dataset with clinical diagnosis was included as the outcome dataset. The exposure and exposure datasets are displayed in Table 1. All datasets used in this MR analysis were based on the European population to ensure that the population was consistent with many other MR analyses [19, 20]. The authors aimed to avoid possible participant overlap bias. The website https://sb452.shinyapps.io/overlap/ [21] was employed to ensure that all dataset overlaps < 10%.

**Table 1:** Information on the datasets used in this MR analysis

Information	Dataset ID in IEUGWAS	Year	Sample size	Cases	Controls	Sex and gender ratio	Age range	Numbers of SNPs
UC 1 dataset	finn-b-K11_UC_STRICT	2021	218,507	2,701	215,806	Males (Proportion NA) and Females (NA)	NA	16,380,466
UC 2 dataset	Ieu-a-32	2015	27,432	6,968	20,464	Males (NA) and Females (NA)	NA	12,255,197
UC 3 dataset	ukb-a-104	2017	337,159	1,795	335,364	Males (NA) and Females (NA)	NA	10,894,596
UC 4 dataset	ukb-d-ULCERNAS	2018	361,194	1,903	359,291	Males (NA) and Females (NA)	NA	10,366,063
Heart failure	ebi-a-GCST90018806	2021	486,160	14,262	471,898	Female proportion of UKB recruitments was 53.8%, Finngen 56.3%.	Mean age of UKB recruitments was 56.8 year old, Fin Finngen 51.8-year-old	24,178,220

UKB: United Kingdom biobank. NA: Not available. The datasets are all available at the IEU website (https://gwas.mrcieu.ac.uk/)

### Selection of genetic instrumental variables (IVs)

The selection of genetic IVs in MR analyses of UC and heart failure must meet the following criteria. First, the genome-wide significance level probability value (*P* value) < 5 × 10^–8^, correlation coefficient (R) ^2^ < 0.001 and the IVs must be within the 10,000 kilobase pairs (kb) threshold distributed independently by clumping single nucleotide polymorphisms (SNPs) [22]. Second, SNPs of the palindrome structure were removed from the IVs. Third, to avoid potential pleiotropy, the PhenoScanner database (version 2; http://www.phenoscanner.medschl.cam.ac.uk/) was used to examine the associations of each candidate IV with confounders [23]. The confounding factors related to the SNPs were carefully deleted only after screening previous MR studies and other high-quality reports of UC and heart failure, including trials and reviews. SNPs that were considered confounding factors were removed [24]. Fourth, the F-statistic calculation was performed following this formula: F = ((sample size - number of IVs − 1)/ number of IVs)*((proportion of the variability of the exposure explained by IVs ^2^)/1 - proportion of the variability of the exposure explained by IVs ^2^) [25, 26]. SNPs of IVs with F-statistic < 10 were recognized as weak IVs and were thus removed [27]. Fifth, the Steiger test filtering method was applied to identify and exclude pleiotropic SNPs [28].

### MR statistical analysis and sensitivity analysis

The UC and heart failure GWASs information including β-coefficient or log odds ratio, 95% CI, effect allele, other allele, *p*-value, effect allele frequency and sample size of subjects were gained. The “Two-Sample-MR”, “MR” and “MR-PRESSO” packages in R software (version 4.3.3; R Project for Statistical Computing, Vienna, Austria) were used for statistical analysis. If exposure ≥ 2SNPs (In most cases), result of the inverse variance weighted (IVW) method (Which assuming that all SNPs are valid instrumental.

variables) was considered the main outcome [29]. The other 4 approaches included MR‒Egger [30], weighted median [31], simple mode and weighted mode were set as auxiliary methods. If only 1 SNP in exposure, Wald (ratio of coefficients) method was applied. The MR‒Egger approach was employed to explore the potential of pleiotropy [32]. Cochran’s Q method was used to analyze heterogeneity. The MR-PRESSO method was used to conduct a global test of heterogeneity and for the identification of horizontal pleiotropy [33]. MR-PRESSO (outlier methods) was applied for sensitivity analyses [34]. In MR analysis, if there are missing values in the data, sensitivity analysis is used to test the stability of missing values on the results by increasing or decreasing the missing values to observe whether the results change. In addition, leave-one-out (LOO) sensitivity analysis was performed to assess whether the causal association was elicited by an individual SNP. Odds ratios (ORs) with 95% confidence intervals (CIs) were used as the effect size measures, and *P* values < 0.05 were considered to indicate statistical significance [35]. If pleiotropy was observed, the Steiger test filtering method was applied to each SNP to conduct sensitivity analysis and to exclude pleiotropic SNPs [28].

### Evaluation of genetic correlation and directionality

Linkage disequilibrium score (LDSC) regression was performed via the R package assay in python software [36] to explore the genetic correlation between the UC datasets and heart failure; this method was used in order to avoid the coheritability of exposure [37].

## Results

### IVs

Each IV had an F index greater than 10, suggesting that weak instrument bias did not affect the results of this study. No confounding factors were found to be related to SNPs based on high-quality literature. The SNPs with palindrome structures were eliminated. Based on the results of MR-PRESSO (outlier methods) rs9891174 in the IEUGWAS ID of ieu-a-32, further rs9891174, was excluded from further analysis. The other 3 IEUGWAS studies did not report outlying SNPs. Additionally, all the SNPs passed the Steiger test, suggesting that none of included SNPs were confounding factors.

### MR results of ulcerative colitis to chronic heart failure

**Fig. 2:**
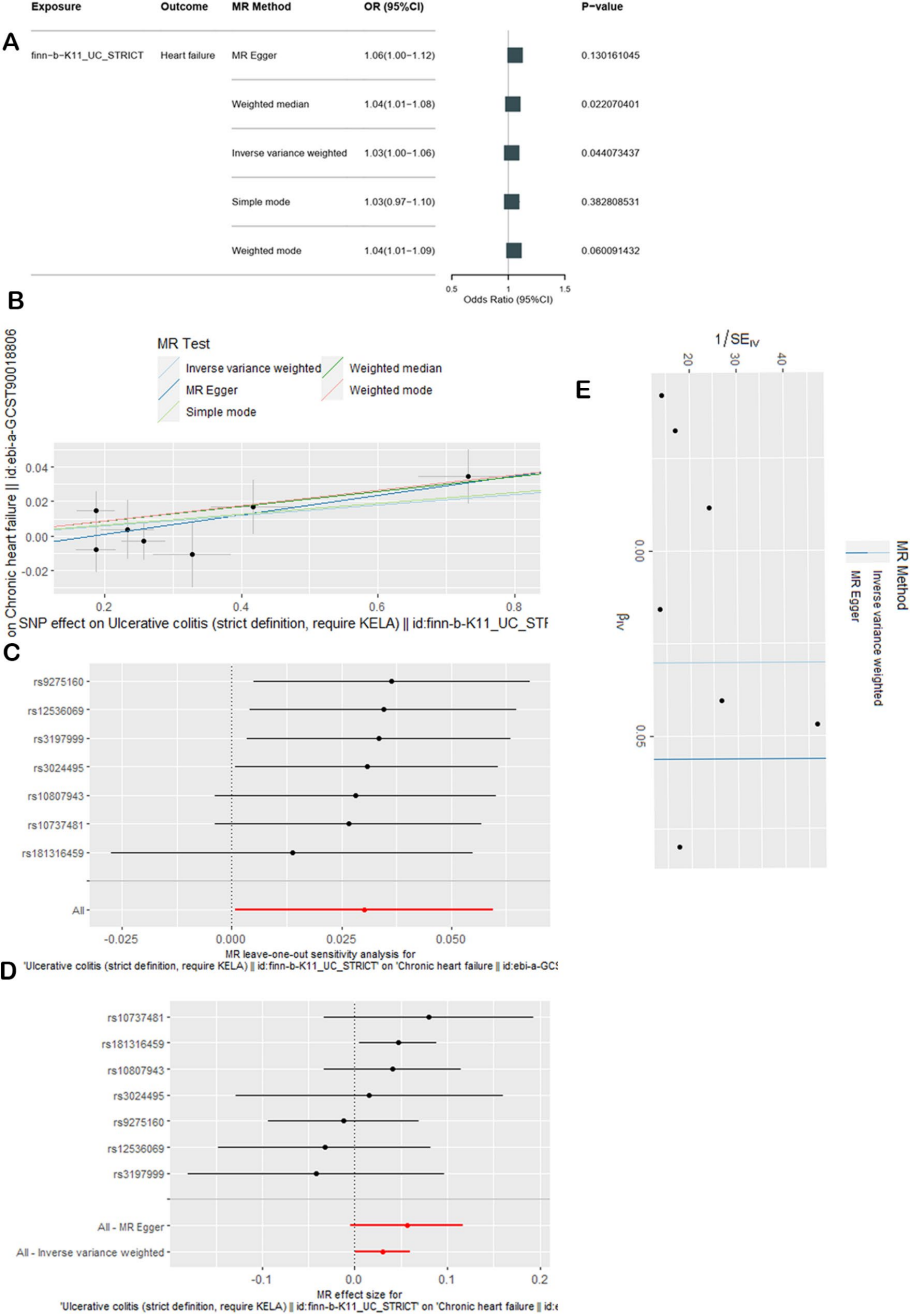
Casual effects of UC on heart failure in the first dataset. (**A**) Effects of UC on heart failure according to the 5 methods. (**B**) SNP effect scatterplot. (**C**) Sensitivity analysis using the leave-one-out method. (**D**) Forest map generated via the MR-Egger and IVW methods. (**E**) Funnel plot of bias

Figure 2A shows the causal effect of UC on chronic heart failure based on the first ulcerative colitis dataset. The IVW method yielded an odds ratio (OR) = 1.03, 95% confidence interval (CI) = 1.01–1.06, *P* = 0.0441 < 0.05 for the effect of ulcerative colitis on chronic heart failure. Among the other 4 MR methods, the weighted mean method showed consistent results, but the other 3 methods (MR egger, weighted mode, simple mode) presented the opposite results. These results are shown in Fig. 2B, which illustrates the positive association between genetically increased odds of promoting chronic heart failure and exposure to ulcerative colitis. According to the main IVW results and one results from an auxiliary method, UC has a certain causal effect on heart failure. There was no evidence of pleiotropy. The MR‒Egger regression intercept estimates were close to 0, and the *P* values were greater than 0.05 (Table 2). Heterogeneity was assessed via the MR‒Egger and IVW methods, which demonstrated that the *P* values were all greater than 0.05 (Table 2). Sensitivity analysis was performed via the LOO method (Fig. 2C) and showed that no individual SNP had led to a change in the results, thus suggesting that the MR estimate was relatively stable. Furthermore, a forest map was constructed to depict the consistency of the IVW method and MR‒Egger method results, which are both on the same side of the 0 value (Fig. 2D), suggesting consistent statistical significance in both 2 method results. The funnel plot (Fig. 2E) was approximately symmetrical, suggesting that there was a low risk of bias.

**Table 2:** Heterogeneity and pleiotropy analysis in this bidirectional MR analysis

Exposure	Outcome	Methods	Heterogeneity	Pleiotropy	
			Q_pval	egger_intercept	pval
UC dataset 1	Heart failure	MR Egger	0.5843	-0.0103	0.3815
		IVW	0.5852		
UC dataset 2	Heart failure	MR Egger	0.1319	-0.0100	0.1063
		IVW	0.0884		
UC dataset 3	Heart failure	MR Egger	0.9099	0.0090	0.4769
		IVW	0.9131		
UC dataset 4	Heart failure	MR Egger	0.6291	0.0143	0.3753
		IVW	0.6112		
Heart failure	UC dataset 1	MR Egger	0.0131	-0.0580	0.4730
		IVW	0.0105		
Heart failure	UC dataset 2	MR Egger	0.8432	0.0377	0.4457
		IVW	0.8101		
Heart failure	UC dataset 3	MR Egger	0.4663	0.0003	0.3494
		IVW	0.4375		
Heart failure	UC dataset 4	MR Egger	0.1635	0.0004	0.3532
		IVW	0.1275		

**Fig. 3:**
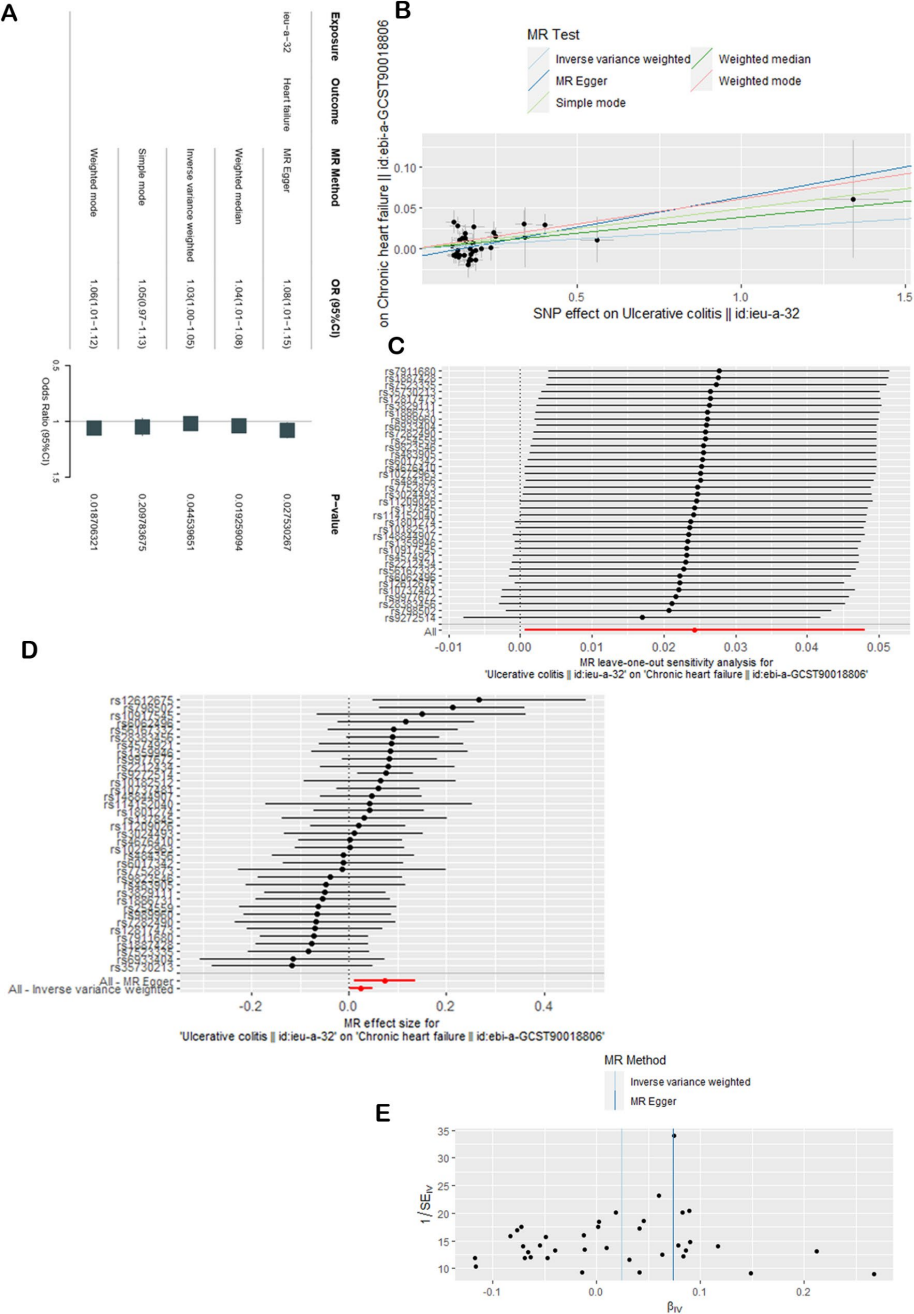
Casual effects of UC on heart failure in the second dataset. (**A**) Effects of UC on heart failure according to the 5 methods. (**B**) SNP effect scatterplot. (**C**) Sensitivity analysis using the leave-one-out method. (**D**) Forest map generated via the MR-Egger and IVW methods. (**E**) Funnel plot of bias

The results of the MR analysis examining the causal effect of ulcerative colitis on chronic heart failure were similar when the second ulcerative colitis dataset was used. The main analysis via the IVW method yielded an OR = 1.03, 95% CI = 1.01–1.05, *P* = 0.0445 < 0.05 for the effect of ulcerative colitis on chronic heart failure. The results of the other 4 methods were largely consistent with those of the IVW method (with the exception of the simple mode method, Fig. 3A). Figure 3B shows the positive association between genetically increased odds of promoting chronic heart failure in patients with ulcerative colitis, suggesting that UC has a causal effect on heart failure. There was no evidence of pleiotropy. The MR‒Egger regression intercept estimates were close to 0, and the *P* values were greater than 0.05 (Table 2). Heterogeneity was assessed via the MR‒Egger and IVW methods, which revealed that the *P* values were all greater than 0.05 (Table 2). Sensitivity analysis was performed via the LOO method (Fig. 3C) and showed that no individual SNP led to changes in the overall results, suggesting that the MR estimate was relatively stable. Furthermore, a forest map was constructed to depict the consistency of the IVW method and MR‒Egger method results, which are both on the same side of the 0 value (Fig. 3D), suggesting consistent statistical significance in both 2 method results. The funnel plot (Fig. 3E) was approximately symmetrical, suggesting that there was a low risk of bias.

**Fig. 4:**
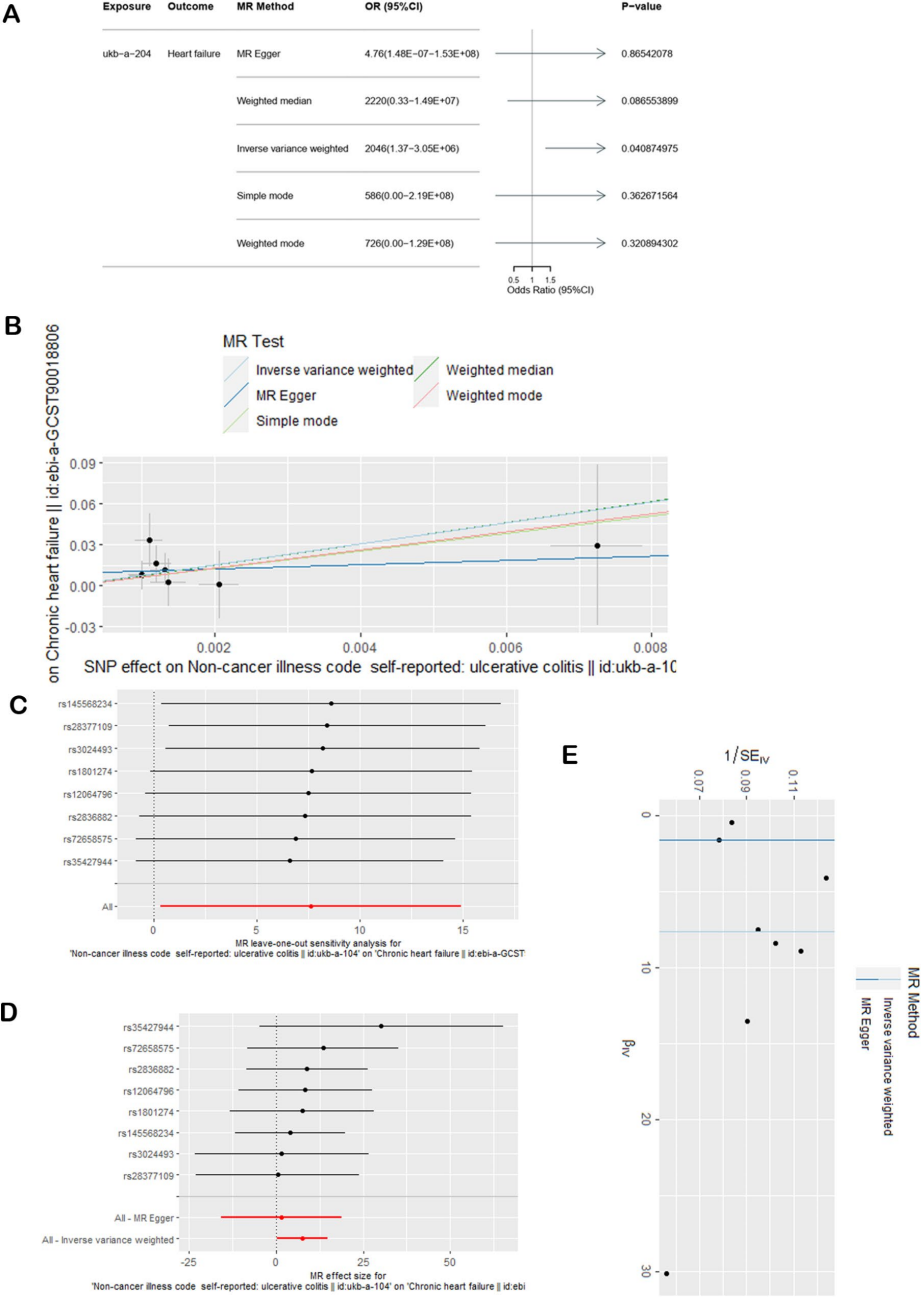
Casual effects of UC on heart failure in the third dataset. (**A**) Effects of UC on heart failure according to the 5 methods. (**B**) SNP effect scatterplot. (**C**) Sensitivity analysis using the leave-one-out method. (**D**) Forest map generated via the MR-Egger and IVW methods. (**E**) Funnel plot of bias

The results of the MR analysis examining the causal effect of ulcerative colitis on chronic heart failure were similar when the third ulcerative colitis dataset was used. The main analysis via the IVW method yielded an OR = 2046, 95% CI = 1.37-3.05E + 06, *P* = 0.0409 < 0.05 for the effect of ulcerative colitis on chronic heart failure. However, the other 4 reference methods yielded different results compared with the IVW method (Fig. 4A). Figure 4B shows the positive association between genetically increased odds of promoting chronic heart failure in patients with ulcerative colitis, suggesting that UC has a causal effect on heart failure. There was no evidence of pleiotropy. The MR‒Egger regression intercept estimates were close to 0, and the *P* values were greater than 0.05 (Table 2). Heterogeneity was assessed via the MR‒Egger and IVW methods, which revealed that the *P* values were all greater than 0.05. Sensitivity analysis was performed via the LOO method (Fig. 4C), which showed that no individuals SNP led to changes in the overall results, thus suggesting that the MR estimate was relatively stable. Furthermore, a forest map was constructed to depict the consistency of the IVW method and MR‒Egger method results, which are both on the same side of the 0 value (Fig. 4D), suggesting consistent statistical significance in both 2 method results. The funnel plot (Fig. 4E) was approximately symmetrical, suggesting few bias in MR analysis.

**Fig. 5:**
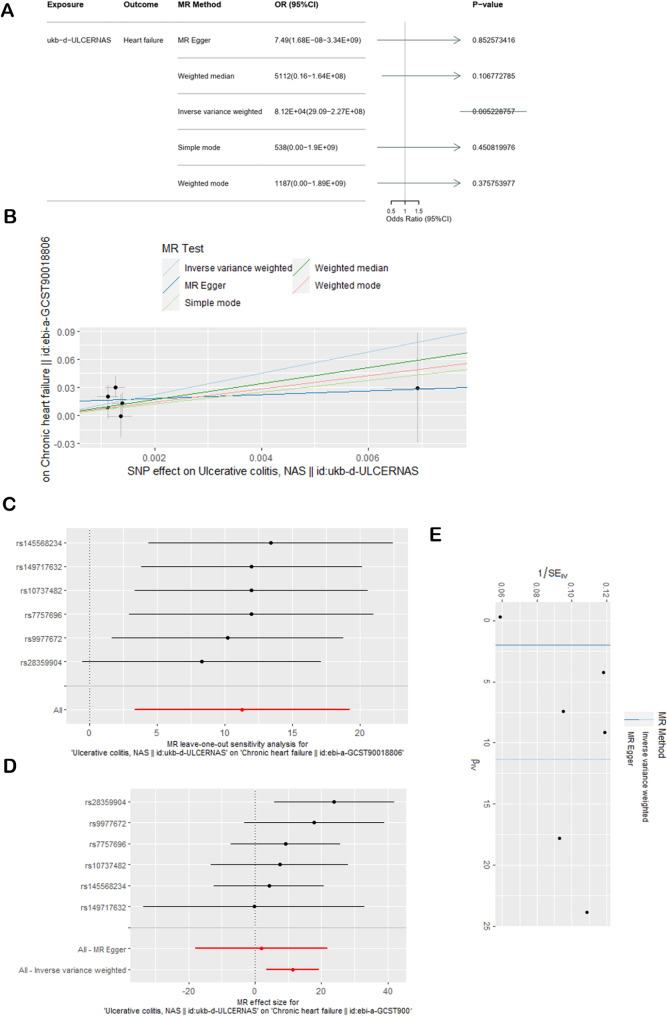
Casual effects of UC on heart failure in the fourth dataset. (**A**) Effects of UC on heart failure according to the 5 methods. (**B**) SNP effect scatterplot. (**C**) Sensitivity analysis using the leave-one-out method. (**D**) Forest map generated via the MR-Egger and IVW methods. (**E**) Funnel plot of bias

The results of the MR analysis examining the causal effect of ulcerative colitis on chronic heart failure were similar when the fourth ulcerative colitis dataset was used. The main analysis via the IVW method yielded an OR = 8.12E + 04, 95% CI = 29.09-2.27E + 08, *P* = 0.0052 < 0.05 for the effect of ulcerative colitis on chronic heart failure. The results obtained via the other 4 reference methods were all different from those of the IVW method (Fig. 5A). However, Fig. 5B still shows a positive association between genetically increased odds of promoting chronic heart failure and ulcerative colitis, suggesting that UC has a causal effect on heart failure. There was no evidence of pleiotropy. The MR‒Egger regression intercept estimates were close to 0, and all the *P* values were greater than 0.05 (Table 2). Heterogeneity was assessed via the MR‒Egger and IVW methods, which yielded *P* values greater than 0.05 (Table 2). Sensitivity analysis was performed via the LOO method (Fig. 5C), which revealed that no individual SNP changed the overall results, thus suggesting that the MR estimate was relatively stable. Furthermore, a forest map was constructed to depict the consistency of the IVW method and MR‒Egger method results, which were both on the same side of the 0 value (Fig. 5D), suggesting consistent statistical significance in both 2 method results. The funnel plot (Fig. 5E) was approximately symmetrical, suggesting few bias in MR analysis.

### Meta-analysis

**Fig. 6:**
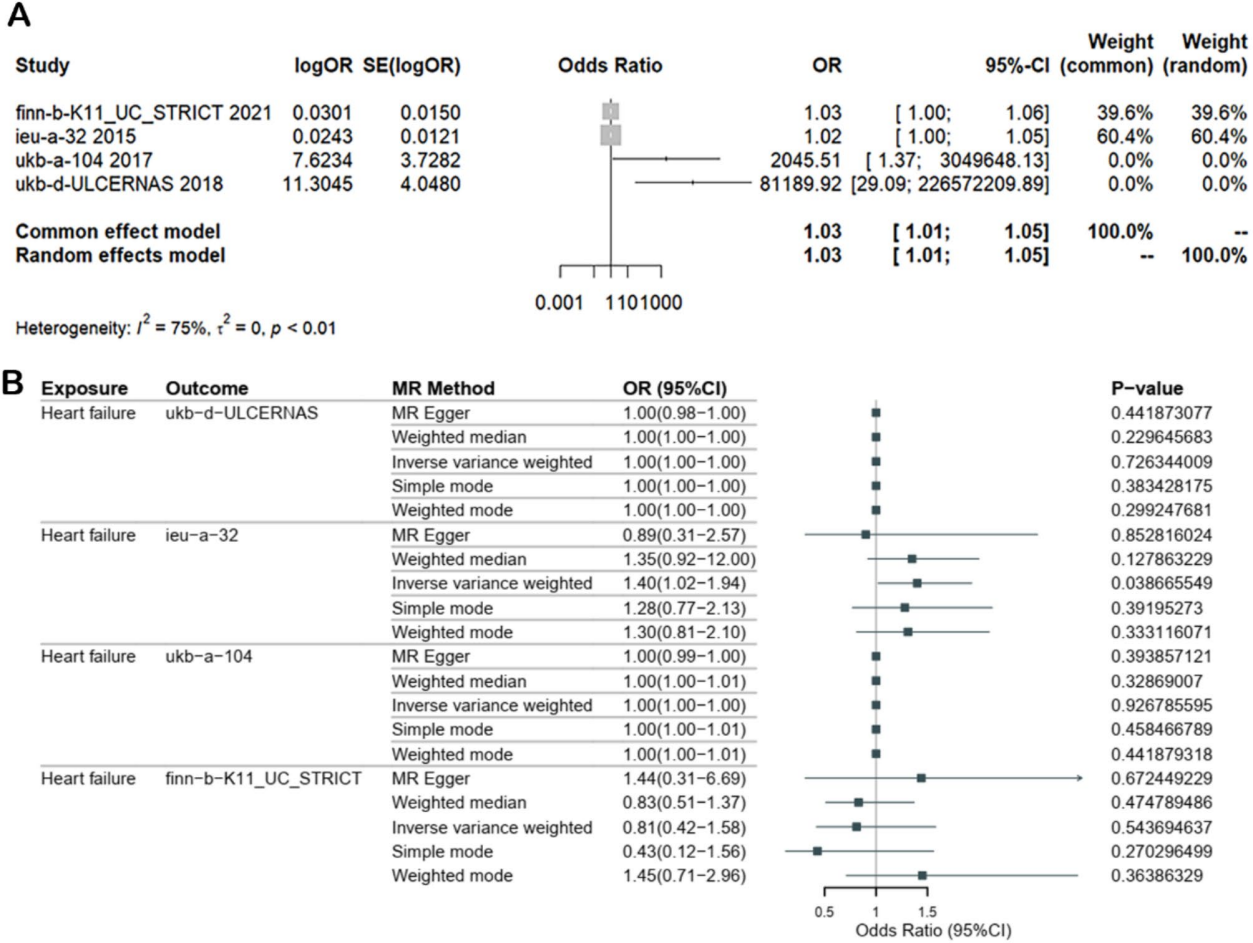
(**A**) Meta-analysis of the pooled effect of 4 MR estimates. (**B**) Reverse MR analysis of heart failure on UC using 4 GWASs

The 4 MR studies in the above section were combined and examined via meta-analysis. As shown in Fig. 6A, there was a statistically significant pooled causal effect of UC on heart failure (odds ratio = 1.03, 95% CI = 1.01–1.05, *P* = 0.0074 < 0.05), with a high degree of heterogeneity (I^2^ = 75%). The pooled effect of the 4 single MR studies was the same as that of a combine effect of 4 single MR studies, which suggested that UC has a causal effect on heart failure and promotes the development of heart failure.

### Reverse MR analysis

As shown in Fig. 6B, reverse MR analysis using UC as the outcomes and heart failure as the exposure revealed no statistically significant causal effect, with *P* values > 0.05 for nearly all 5 statistical methods and all 4 UC datasets (there was a *p* value of 0.039 < 0.05 for one analysis, which was performed using the IVW method to examine the effect of heart failure on UC based on the data from the ieu-a-32 dataset). There results suggest that heart failure is not a risk factor for UC and cannot promote the development of UC. The sensitivity analysis revealed a lack of pleiotropy (all *P* values > 0.05, displayed in Table 2). Heterogeneity was only observed in the UC1 dataset (*P* = 0.0105 < 0.05 for the IVW method and *P* = 0.0131 < 0.05 for the MR‒Egger method, as displayed in Table 2).

### Genetic correlation via the LDSC statistic

The LDSC statistics revealed weak genetic correlations between the UC 1 dataset and heart failure ((Genetic correlation (Rg) = -0.6992, Genetic correlation standard error (Rg_Se) = 1.5788, Genetic correlation *p* value (Rg_*P*) = 0.6578 > 0.05), the UC 2 dataset and heart failure (Rg = -0.9199, Rg_Se = 1.1325, Rg_*P* = 0.4166 > 0.05), and the UC 4 dataset and heart failure dataset (Rg = -0.1595, Rg_Se = 0.9751, Rg_*P* = 0.8701 > 0.05). These findings indicated that MR estimates of the effect of UC on heart failure were not biased by shared genetic components. UC dataset 3 was not included in this analysis due to lack of data.

## Discussion

In this comprehensive MR analysis, UC was found to have a statistically significant (*p* value < 0.05) casual effect on heart failure. Several methods were applied in this MR analysis, such as bidirectional MR, strict instrumental variable control, LDSC, and meta-analysis.

Owing to the random distribution of alleles passed down from parents to their offspring, MR studies can utilize genetic variation as instrumental variables to conduct “natural randomized controlled studies,” thereby enabling the estimation of causal associations between modifiable risk factors (exposure) and disease outcomes [38]. MR studies must meet 3 core assumptions [39]: (1) genetic instruments (IVs) should be correlated with exposure (the relevance assumption); (2) IVs are not associated with any potential confounders of the exposure‒outcome association (independent assumption); and (3) IVs are not related to the outcome only via the method of exposure (exclusion restriction assumption), as shown in Fig. 1. The IVW method is an ideal estimation method; therefore, it is commonly used as the primary MR method. The IVW method is an effective analysis under the basic premise that all genetic variants are effective instrumental variables [29]. It has strong causal relationship detection ability. However, the IVW method specifically requires that genetic variation affects the target outcome only through the exposure in the study. Therefore, the results of the other 4 methods need to be used as auxiliary references to test the reliability and stability of the results. The ORs in the MR study obtained via the IVW method and the other 4 methods were used to measure the difference in the performance of genetic traits between the two diseases. When the OR is greater than 1, the probability of the occurrence of exposure to the outcome is positively correlated. The OR was calculated on the basis of the beta value of exposure. Beta coefficients are representative per allele log OR [40]. The OR calculation needs to refer to other values, such as the 95% CI and *P* value. Even if the OR value is close to 1, if the *p* value is < 0.05, this effect may also be statistically significant. In addition, meta-analysis was conducted by pooling the results of several single MR studies, thereby increasing the robustness of the causal effect. In this study, the results of the IVW methods from 4 single MR studies and the pooled effect of the 4 MR studies in the meta-analysis both revealed a statistically significant causal association between UC and heart failure.

Furthermore, one of the hypotheses of MR analysis is that IVs can only affect the outcome through exposure. If IVs do not directly affect the outcome through affecting the exposure, it violates the idea of MR. Therefore, it is necessary to test whether there is pleiotropy in the causal inference between exposure and outcome [41]. The core of the MR-presso method test is to calculate the IVW result after removing each SNP, calculate the sum of the residual square of the effect of this SNP and the IVW result, and finally add the sum of the residual square of all SNPs. The larger the value is, the more significant the pleiotropy. In the LOO method for sensitivity anlaysis, if the pooled outcomes change markedly after excluding an individual SNP, the MR results are considered to be sensitive to this SNP [42].

There are several reports that support the potential biological association between UC and heart failure. A Danish nationwide cohort study revealed that IBD, which includes UC and Crohn’s disease, is related to a high risk of heart failure [14]. A meta-analysis published in 2018 suggested that there was a positive association between IBD and a higher risk of cardiovascular disease incidence [43]. Furthermore, a previous review overlooked IBD as a contributor to atherosclerotic cardiovascular disease [44], especially in women and young adults. IBD is a colon disorder associated with increased levels of proinflammatory cytokines, including tumor necrosis factor-a, interleukin (IL)-1b, IL-2, and IL-6. The oxidative stress levels also changed. This leads to changes in the phenotype of smooth muscle cells and triggers a series of events, ultimately leading to atherosclerosis or heart disease [45]. Previous studies have revealed that, compared with prolonged corticosteroid treatment, anti- tumor necrosis factor -α therapy is linked to a decreased incidence of cardiovascular events in IBD patients and reduced aortic stiffness [46, 47]. IBD patients, particularly younger patients, should also be screened for atherosclerotic cardiovascular disease risk factors to reduce the risk of cardiovascular events [48].

These reports revealed the strong association between UC and heart failure. Our research is consistent with the above reports and meta-analyses [43] but is not entirely consistent with the most recent MR study on IBD and heart disease [49]. In that study, the researchers did not find a causal association between IBD and elevated risk of cardiometabolic diseases. However, their study included data from 2006 to 2010, with only 3635 UC samples and 4862 heart failure samples [49]. This may have contributed to the difference between their MR results and our MR study. Compared with their previous MR study, our study focused more on the role of UC in heart failure, using more recent GWAS data published in 2021. Furthermore, we used 4 UC datasets containing a total of different years, thus yielding a sample size that was approximately three-fold larger than that of the previous study. Finally, the pooled effect was estimated via a meta-analysis, thus yielding a more reliable MR result.

This MR study has several strengths and limitations. First, this study provides new evidence-based MR results with larger sample sizes than most recent MR studies. Second, there are many strict parameters for this MR to ensure robust MR estimates, such as the selection of instrumental variables and sensitivity analysis. In addition, in our preliminary experiment, we found a statistically significant association between UC and many heart failure datasets; however, some results contained fewer SNPs, thus making them unsuitable for MR analysis. A limitation was that although MR studies using summary level data from meta-analysis GWAS datasets online have become common practice in the field [50, 51], the characteristics of the study population, such as the age range and sex ratio, were limited in many GWAS datasets. Another limitation was that although MR studies can investigate the possible causal correlation from exposure to outcome, the lack of proper other influencing factors in life means that the observed correlation may not 100% happens. The incidence of outcome disease is still affected by additional factors in many cases. Our study mainly revealed that UC is a risk factor for heart failure.

## Conclusion

This MR study provided genetic evidence that supported the statistically significant causal association between UC and heart failure. Although there were some limitations, these findings could help UC patients take precautions against chronic heart failure in the future.

## Electronic supplementary material

Below is the link to the electronic supplementary material.

Supplementary Material 1

## Data Availability

No datasets were generated or analysed during the current study.

## Abbreviations

GWAS: Genome-wide association study
UC: Ulcerative colitis
HF: Heart failure
IBD: Inflammatory bowel disease
MR: Mendelian randomization
IVs: Instrumental variables
*P* value: Probability value
SNPs: Single nucleotide polymorphisms
ORs: Odds ratios
LOO: Leave-one-out
CIs: Confidence intervals
IVW: Inverse variance weighted
kb: Kilobase pairs
LDSC: Linkage disequilibrium score
